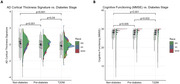# Alzheimer disease ATN imaging biomarkers across type 2 diabetes mellitus stages in diverse populations

**DOI:** 10.1002/alz.088263

**Published:** 2025-01-09

**Authors:** Malavika Pillai, Beau Ances, Sid E. O'Bryant, Karin L. Meeker

**Affiliations:** ^1^ Washington University in St. Louis School of Medicine, St. Louis, MO USA; ^2^ University of North Texas Health Science Center, Fort Worth, TX USA

## Abstract

**Background:**

Type 2 diabetes mellitus (T2DM) is a risk factor for Alzheimer disease (AD) and disproportionately impacts under‐represented groups including Mexican Americans/Hispanics (MAs) and African Americans/Blacks (AAs) compared to non‐Hispanic Whites (NHWs). However, it remains unclear how amyloid, tau, and neurodegeneration (AT(N)) AD imaging biomarkers and cognitive functioning differ across diabetic stages including non‐diabetes, pre‐diabetes, and T2DM in a diverse community‐based cohort.

**Method:**

Data were obtained from the well‐characterized Health and Aging Brain Study: Health Disparities (HABS‐HD) cohort, including MAs (n=612), AAs (n=676), and NHWs (n=725) with clinical, amyloid and tau positron emission tomography (PET), and cortical thickness measures from magnetic resonance imaging (MRI) scans. Diabetic stage was categorized by HbA1c values: non‐diabetes=HbA1c<5.7%; pre‐diabetes=HbA1c 5.7‐6.4%; and T2DM=HbA1c>6.4%. Differences in continuous measures of AT(N) imaging biomarkers and cognitive functioning (Mini‐Mental State Examination) across diabetes stage were determined using multivariate analyses of covariance (MANCOVAs). The interactive effect of diabetes stage and ethno‐racial group on AT(N) biomarkers and cognitive functioning was also assessed. Age, sex, white matter hyperintensity volume, and Clinical Dementia Rating (CDR) Sum of Boxes were included as covariates.

**Result:**

The AD cortical thickness signature was significantly decreased in pre‐diabetes compared to non‐diabetes (p=0.001), and T2DM compared to non‐diabetes (p<0.001) and pre‐diabetes (p=0.04). Cognitive functioning was also decreased in pre‐diabetes compared to non‐diabetes (p=0.005), and T2DM compared to non‐diabetes (p<0.001) and pre‐diabetes (p=0.002). Amyloid and tau PET did not significantly differ across diabetes stages (p’s>0.05). Amyloid burden was significantly increased in NHWs compared to MAs and AAs (p’s<0.001). Cognitive functioning was significantly decreased in MAs compared to AAs and NHWs, and in AAs compared to NHWs (p’s<0.001). Tau PET and cortical signature did not significantly differ by group (p’s>0.05). MAs and AAs had higher incidences of pre‐diabetes/T2DM, but no interactions between diabetes stage and ethno‐racial group were observed for AT(N) biomarkers and cognitive functioning.

**Conclusion:**

AD‐related neurodegeneration and cognitive functioning worsen in the earliest stages of diabetes. Although under‐represented groups have higher T2DM rates, the deleterious effects of higher HbA1c levels are ubiquitous across diverse populations. Treatment of T2DM may alleviate downstream AD decline and should be considered in AD clinical settings, studies, and trials.